# Combined Id1 and Id3 Deletion Leads to Severe Erythropoietic Disturbances

**DOI:** 10.1371/journal.pone.0154480

**Published:** 2016-04-29

**Authors:** Qingshi Zhao, Corey Chang, J. Patrick Gonzalez, Kamal Alzahrani, Jessica L Button, Diego Fraidenraich

**Affiliations:** Department of Cell Biology and Molecular Medicine, Rutgers Biomedical and Health Sciences, New Jersey Medical School, Newark, New Jersey, United States of America; Emory University, UNITED STATES

## Abstract

The Inhibitor of DNA Binding (Id) proteins play a crucial role in regulating hematopoiesis and are known to interact with E proteins and the bHLH family of transcription factors. Current efforts seek to elucidate the individual roles of Id members in regulating hematopoietic development and specification. However, the nature of their functional redundancies remains elusive since ablation of multiple Id genes is embryonically lethal. We developed a model to test this compensation in the adult. We report that global *Id3* ablation with Tie2Cre-mediated conditional ablation of *Id1* in both hematopoietic and endothelial cells (Id cDKO) extends viability to 1 year but leads to multi-lineage hematopoietic defects including the emergence of anemia associated with defective erythroid development, a novel phenotype unreported in prior single Id knockout studies. We observe decreased cell counts in the bone marrow and splenomegaly to dimensions beyond what is seen in single Id knockout models. Transcriptional dysregulation of hematopoietic regulators observed in bone marrow cells is also magnified in the spleen. E47 protein levels were elevated in Id cDKO bone marrow cell isolates, but decreased in the erythroid lineage. Chromatin immunoprecipitation (ChIP) studies reveal increased occupancy of E47 and GATA1 at the promoter regions of *β-globin* and *E2A*. Bone marrow transplantation studies highlight the importance of intrinsic Id signals in maintaining hematopoietic homeostasis while revealing a strong extrinsic influence in the development of anemia. Together, these findings demonstrate that loss of Id compensation leads to dysregulation of the hematopoietic transcriptional network and multiple defects in erythropoietic development in adult mice.

## Introduction

The Inhibitor of DNA binding (Id) proteins are a family (Id1-4) of helix-loop-helix (HLH) nuclear factors that bind and sequester the ubiquitous basic HLH (bHLH) E proteins. This binding interaction prevents E proteins from heterodimerizing with tissue-specific bHLH partners. Without E proteins, the tissue-specific bHLH proteins do not bind to E-boxes in the promoter and enhancer regions of target genes and transcription does not proceed, resulting in marked dysregulation of a vast spectrum of cellular events, including proliferation and differentiation [1–12]. The Id genes are expressed in a variety of tissues during development with partially overlapping patterns and functionality. In the adult, expression of Id1 and Id3 becomes confined to discrete structures [13–15] such as the endothelial and hematopoietic compartments [16–23].

Id1 and Id3 are known to play important roles in regulating hematopoiesis. Id1 promotes cell proliferation and regulates myeloid, erythroid and B-cell differentiation [24–29]. Id3 regulates lymphocyte development, including thymocyte and B-lymphocyte proliferation and differentiation [30–35]. As Id members demonstrate the capacity to compensate functionally, current single knockout studies are limited in the ability to achieve a functional ablation. In addition, an important aspect of the Id mode of action is non-cell autonomous, via secretion of paracrine factors [36]. An assessment of the combined impact of the loss of *Id1* and *Id3* genes in hematopoiesis has been precluded by the lethality of *Id1/Id3* double knockout (Id DKO) embryos [36,37].

To address the combined role of Id1 and Id3 in hematopoiesis, we circumvented embryonic lethality by ablating *Id3* globally and conditionally ablating *Id1* in both the endothelium and hematopoietic compartments [16,36]. We chose Tie2 as a driver of Cre/lox recombination because *Tie2* is expressed at 9.5 days post coitum (9.5 dpc) in hematopoietic and endothelial cells, an important component of the hematopoietic niche [38–40].

In this study we report that this severe model of Id ablation leads to roughly 70% postnatal survival with lethality by 12 months. Findings unveiled unsuspected defects in maturation of the erythroid lineage in the bone marrow and spleen that ultimately lead to anemia.

## Materials and Methods

### Mouse colonies and genotyping

Id1^F/F^Id3^-/-^ and Id1^F/-^Id3^-/-^ (Id control) and Tie2Cre^+^Id1^F/F^Id3^-/-^ and Tie2Cre^+^Id1^F/-^Id3^-/-^ (Id cDKO) mice were generated as described previously [16]. Mice were genotyped by PCR on freshly isolated DNA from tail tips using published primers for Id1 wild type, Id1 mutant, Id1 flox, Id3 wild type, Id3 mutant and Tie2Cre [16]. Ub-GFP transgenic WT C57Bl/J6 mice served as bone marrow donors for forward bone marrow transplantation experiments and as bone marrow recipients for reverse bone marrow transplantation experiments. R26LacZR26 mice, B6.129S4-Gt(ROSA)26Sor^tm1Sor^/J, used for verification of Cre/loxP-mediated recombination, were purchased from The Jackson Laboratory. All animal experiments were approved by the IACUC of Rutgers New Jersey Medical School and performed in accordance with relevant guidelines and regulations. In addition to marked splenomegaly and hematopoietic defects, Id cDKO mice develop dilated fibrotic cardiomyopathy [16]. Clinical signs of pain/distress include weakness and poor responsiveness to external stimuli. Mice were monitored on a daily basis. Mice were euthanized if signs of pain or distress were observed. Mice were euthanized by carbon dioxide inhalation followed by cervical dislocation, carbon dioxide inhalation followed by decapitation or carbon dioxide inhalation to effect.

### Cell preparation and Flow cytometry

Complete blood count (CBC) analysis was performed on freshly harvested peripheral blood cells (PBCs) by IDEXX RADIL Laboratory Animal Diagnostics and reticulocytosis was determined by analysis of blood smears. Total nucleated bone marrow and spleen cell counts were determined by hemacytometer counting. For flow cytometry analysis, single-cell suspensions were prepared from bone marrow (BM), spleen and thymus in Dulbecco phosphate-buffered saline (PBS) supplemented with 0.1% fetal bovine serum. Cells were stained with monoclonal antibodies labeled with different dyes that recognize specific-lineage anti-Gr-1, anti-CD11b and anti-F4/80 for myeloid cells; anti-B220 and anti-IgM for B cells; anti-CD3, anti-CD4 and anti-CD8 for T cells; and anti-TER119 and anti-CD71 for erythroid cells. These antibodies were purchased from eBioscience. To analyze hematopoietic stem cell (HSC) populations, Lin, Sca1 and cKit antibodies were utilized on bone marrow isolates. These antibodies were purchased from Abcam. Stained cells were analyzed with a FACSCalibur (BD).

### Mouse Colony Forming Unit (CFU) Assay using Methylcellulose-based Media

Total bone marrow cells were freshly isolated by flushing the femurs and tibias of mice from each study group using a 27G½PrecisionGlide needle (Becton-Dickinson Cat#305109) with Iscove’s Modified Dulbecco’s Medium (IMDM) (Invitrogen Cat#12440) supplemented with 2% fetal bovine serum (FBS) (Invitrogen Cat#26140). Debris was removed by straining the cells through a 40μm nylon cell strainer (BD Falcon Cat#352340). Cells were centrifuged at 2000 rpm (900 x g) for 10 mins at 4°C. Cells were subsequently resuspended and counted using a hemacytometer (Bright-Line Cat#420331) and approximately 5x10^5^ cells (suspended in 300μL IMDM/2% FBS) were isolated from each study group. 3 mL aliquots of mouse methycellulose complete (R&D Cat#HSC007) media were thawed on ice. Cells were added to the methylcellulose media, vortexed vigorously and left undisturbed on ice for 20 mins to allow for air bubbles to escape. The methylcellulose/cell mixture was then plated on a 6-well cell culture plate at approximately 1.1mL/well on a 6 well plate (BD Biosciences Cat#351146) using a 16G1½ PrecisionGlide needle (Becton-Dickinson Cat#305198). A 0.5mm grid was drawn on the bottom of each well for counting. Two of the wells on each plate were filled with 10mL of sterile water to ensure proper humidity. Cells were incubated for 12 days at 37°C in a 5% CO_2_ humidified incubator and colonies (BFU-E, CFU-M, CFU-G, GFU-GM, GFU-GEMM) were counted at days 8, 10 and 12. Care was taken to minimize disturbance of the plate during the observation period to avoid shifting of colonies.

### Tube Formation Assay

Human umbilical vein endothelial cells (HUVECs) (Lonza) were used to test the effects of serum on tubulogenesis from different genotypic mice. 5x10^4^ HUVECs (between 2–5 passages) were seeded onto growth factor reduced (GFR) Matrigel (BD Bioscience Cat#354230) on 4-well BD Falcon CultureSlides (Cat#354114). Cells were incubated in the presence of media containing 2% mouse serum from different genotypic groups. After 12 hour incubation (37°C, 5% CO₂), newly formed vessels were imaged using an Olympus 1X51 microscope and analyzed for total number of tubes, tube length and number of nodes. Tube length was measured using ImageJ software.

### Bone Marrow (BM) transplants

Two to three month old recipient mice, WT GFP transgenic mice and Id cDKO-GFP mice (control) as recipients for reverse BM transplants and Id cDKO mice and WT mice (control) as recipients for forward BM transplants, were lethally irradiated with a dosage of 11Gy using a Cs-137 source. On the same day, 2x10⁶ BMCs, from age-matched WT GFP transgenic mice as donor for forward BM transplants (FBMTs) and from Id cDKO mice as donor for reverse BM transplants (RBMTs), were retro-orbitally injected into the recipient mice. Age-matched control mice were irradiated and monitored for irradiation-induced lethality. Animals were monitored daily for signs of morbidity. Reconstitution efficiency in blood cells was determined via flow analysis of the GFP marker. Animals were sacrificed 3–4 months after transplantation.

### Histology, immuno-, X-gal, Prussian Blue, TRAP-staining

Whole embryos were collected at embryonic day E13.5, tissues were cryo-embedded and sectioned, sections were immunostained with primary antibodies for Id1 (Biocheck), as previously described [16].

Adult spleen was fixed with 4% paraformaldehyde (PFA) and embedded with paraffin or fresh spleen tissue were directly OCT embedded for cryo-section. Paraffin sections of spleen and femurs were Hematoxylin and Eosin (H&E) stained for morphological analysis. Cryo-sections of spleen were immunostained with color-labeled anti-F4/80, anti-CD29, anti-TER119, anti-B220 and anti-CD3 monoclonal antibodies after 1 minute fixation with cold acetone and DAPI counterstained as a nuclear background. Visualization of fluorescence was performed using a Nikon Eclipse 80i microscope with NIS Elements Imaging Software. All immunofluorescence antibodies were purchased from eBioscience. X-gal staining was performed on freshly isolated cells from the bone marrow, blood, spleen and thymus of adult mice. X-gal staining solution contained anhydrous X-gal (1 mg/ml) in 1X PBS buffer containing 5 mM potassium ferricyanide, 5 mM potassium ferrocyanide, and 2 mM MgCl_2_ overnight at 37°C.

Adult mouse femurs were cleaned of muscle and fixed for three days with 4% PFA and then treated with 14% ethylenediaminetetraacetic acid (EDTA) for 4 days decalcification, soaked in 30% sucrose in PBS overnight, OCT embedded and cryo-sectioned to a thickness of 6 μm. Tartrate-resistant alkaline phosphatase stain (TRAP) was performed by first incubating in 2% (w/v) Napthol-Ether solution for 1 hr at 37°C, followed by incubating in equimolar amounts of 4% (w/v) sodium nitrite solution and 5% (w/v) pararosaniline dye in basic stock incubation solution for 3–5 mins at 37°C until the desired intensity was achieved, rinsed in 3 changes of distilled water and counterstained with 0.02% Fast Green solution for 45 secs before dehydration and mounting. Basic stock incubation solution consisted of 0.92% (w/v) anhydrous sodium acetate, 1.14% (w/v) dibasic dihydrate sodium tartrate and 0.28% (v/v) glacial acetic acid in distilled water. The pH of the solution was subsequently adjusted between 4.7–5.0 with 5 mM sodium hydroxide.

For Prussian blue staining, paraffin sections of adult spleen were deparaffinized and hydrated to distilled water. Slides were immersed in equal parts 20% (v/v) hydrochloric acid and 10% (w/v) potassium ferrocyanide for 20 mins and counterstained with nuclear fast red for 5 mins before dehydration and mounting. Nuclear red fast solution was prepared with 0.1% (w/v) nuclear fast red and 5% (w/v) aluminum sulfate in distilled water.

Reticulocyte staining on peripheral blood smears was performed according to manufacturer’s protocol using ACCUSTAIN Reticulocyte Stain (Sigma).

### Quantitative polymerase chain reaction

Total RNA was extracted (RNeasy, QIAGEN) and 1-μg total RNA was reverse transcribed using Superscript III reverse transcriptase (Invitrogen). After reverse transcription (RT), the cDNA was used for quantitative PCR (40 cycles of a 10 s-step at 95°C and a 1 min-step at 60°C) with SybrGreen (Applied Biosystems) on a 7300 Sequence Detector (Applied Biosystems). Values are reported per level of β-actin, used as a housekeeping gene. Sequences of primers used are provided below:

*β-actin* s-5’-AGCCATGTACGTAGCCATCC-3’ as-5’-CTCTCAGCTGTGGTGGTGAA-3’; *E2A* s-5’-CATCCATGTCCTGCGAAGCCA-3’ as-5’-TTCTTGTCCTCTTCGGCGTC-3’; *Epo* s-5’-CCACCCTGCTGCTTTTACTC-3’ as-5’-CTCAGTCTGGGACCTTCTGC-3’; *Zfpm1* s-5’-CTACCCCAATGAGGGTGTCT-3’ as-5’-AGAGGATGTCCCTTGTGGTG-3’; *Lmo2* s-5’-TGGATGAGGTGCTGCAGATA-3’ as-5’-GGATGCACAGAGACCATCCT-3’; *Gata1* s-5’-GAAGGGAATGATTGTCAGCA-3’ as-5’-TTCCTCGTCTGGATTCCATC-3’; *Gata2* s-5’-AGACGACAACCACCACCTTA-3’ as-5’-TCCTTCTTCATGGTCAGTGG-3’; *Scl* s-5’-TATGAGATGGAGATTTCTGATG-3’ as-5’-GCTCCTCTGTGTAACTGTCC-3’; *PU1* s-5’-TGGAAGGGTTTTCCCTCACC-3’ as-5’-TGCTGTCCTTCATGTCGCCG-3’; *β-globin* s-5'-ATGGTGCACCTGACTGATGCT G-3' as-5'-GGTTTAGTGGTACTTGTGAGCC-3'

### Western Blot Analysis

Isolated bone marrow cells were washed in 1X PBS and homogenized in lysis buffer containing 50mM TrisCl pH7.5, 150mM NaCl, 1mM sodium pyrophosphate, 1mM NaF, 1mM benzamidine, 5mM sodium orthovanadate, and 0.5% (v/v) NP-40. Lysis buffer was supplemented with protease inhibitor cocktail (Sigma) prior to homogenization. Protein homogenates were loaded onto 10% SDS PAGE Gel and transferred overnight in 4°C at 25 mV onto a nitrocellulose membrane. After blocking, purified mouse anti-human E2A antibody (BD Pharmingen) was applied at dilution of 1:400 in 1% milk solution overnight in 4°C. Mouse anti-GAPDH antibody (Sigma) was applied at dilution 1:10,000 in 1% milk solution overnight in 4°C.

### Chromatin Immunoprecipitation (ChIP)

Briefly, freshly isolated bone marrow cells were fixed with 1% PFA for 15 mins at room temperature. Fixation was stopped by addition of 2.5M glycine solution for 5 mins at room temperature. Cells were subsequently washed and resuspended in chilled PBS-Igepal (Sigma). Cell pellets were resuspended in sonicating buffer (50mM HEPES pH 7.9, 140mM NaCl, 1mM EDTA pH 8, 1% Triton X-100, 0.1% Na-deoxycholate, 0.1% SDS, 0.5mM PMSF), sonicated and the supernatant was collected. After preclearing the lysate with Protein G beads for 2 hrs at 4°C, the supernatant was collected following centrifugation and a portion of the sample was saved for input. Chromatin was isolated by first treating a sample portion with Proteinase K overnight at 65°C and through subsequent phenol/chloroform extraction and glycogen precipitation. DNA concentration was measured and shearing was assessed on a 2% gel. 100 μg of chromatin was then incubated overnight at 4°C with either purified mouse anti-human E47 (BD Pharmingen) for E47 ChIP or GATA1 (M-20:sc-1234) (Santa Cruz) for GATA1 ChIP. Samples were then incubated with Protein G beads for 2 hrs at 4°C. After several washes with sonication buffer, wash buffers and Tris-EDTA (TE) buffer, the beads were incubated with elution buffer (50mM Tris pH8, 1mM EDTA, 1% SDS, 50mM NaHCO_3_) for 10 mins at 65°C. Following centrifugation, the eluate was collected and 4M NaCl was added to both the sample eluate and the input. Sample and input were incubated for 5 hrs at 65°C. Sample and input were then treated with 10mg/mL DNase free RNAse A and incubated for 1 hr at 37°C. 0.5M EDTA and Proteinase K were then added after which the sample and input were incubated for 2 hrs at 42°C. The sample and input were then extracted twice with phenol/chloroform/isoamyl alcohol after which glycogen, 3M Na-acetate and ethanol were added. The sample and input were then vortexed and precipitated overnight at -20°C. After centrifugation and 75% ethanol washes, the pellets were resuspended in water followed by PCR analysis. Sequences of primers used are provided below:

*E2A* promoter s-5’-ATCCCATCCCGACTTTG ATA-3’ as-5’-CGGGATTCTCAGAGCAAGAG -3’; *β-globin* promoter s-5’-AAGCCTGATTCC GTAGAGCCACAC-3’ as-5’-CCCACAGGCA AGAGACAGCAGC-3’

### Proteomic profile

Bone marrow isolates from WT and Id cDKO mice were obtained for global protein identification and quantitation. The extracted proteins were first acetone precipitated and washed with cold acetone several times to remove the salt and primary amine. The resulting proteins were resuspended in solution containing 7M urea, 100mM TEAB and 1X protease inhibitor cocktail. An aliquot of proteins from each sample were run on a SDS-PAGE. The analysis was continued using the 8-plex iTRAQ approach. The protein concentration was measured by Bradford assay. 55μg of protein from each sample were used for the iTRAQ experiment. The proteins were digested by trypsin followed by 8-plex iTRAQ labeling. The resulting peptides were separated by SCX. A total of 20 fractions were collected and further analyzed by RPLC-MS/MS on Orbitrap velos instrument. The MS/MS spectra were searched against Swissprot mouse database using both Mascot and Sequest search engine. Data were analyzed using Ingenuity Pathway software.

For serum proteomic analysis, serum samples were isolated from mice. After serum dilution, the Qproteome Murine Albumin Depletion Kit (Qiagen) was utilized to remove excess albumin from each sample as per manufacturer’s protocol. Samples were loaded onto an SDS-PAGE gel. Gels were stained with Coomassie Brilliant Blue dye and in-gel trypsin digestion was performed. The resulting peptides were C18 desalted and directly analyzed by LC-MS/MS on Q Exactive instrument. The MS/MS spectra were searched against a Swissprot mouse database using MASCOT (V.2.3) search engines on Proteome Discoverer (V1.4) platform. The protein false discovery rate was less than 1%. The protein relative quantitation between Id cDKO and WT was calculated based on spectra counting method and a corresponding p-value was also determined.

### Data analysis

Results are presented as mean ± s.e.m. or as a range. Statistical comparison was performed with nonparametric two-tailed unpaired analysis of variance. All experiments were independently repeated at least 3 times. A probability value of < 0.05 was considered to be statistically significant.

## Results

### Id cDKO mice die prematurely

**Fig 1:**
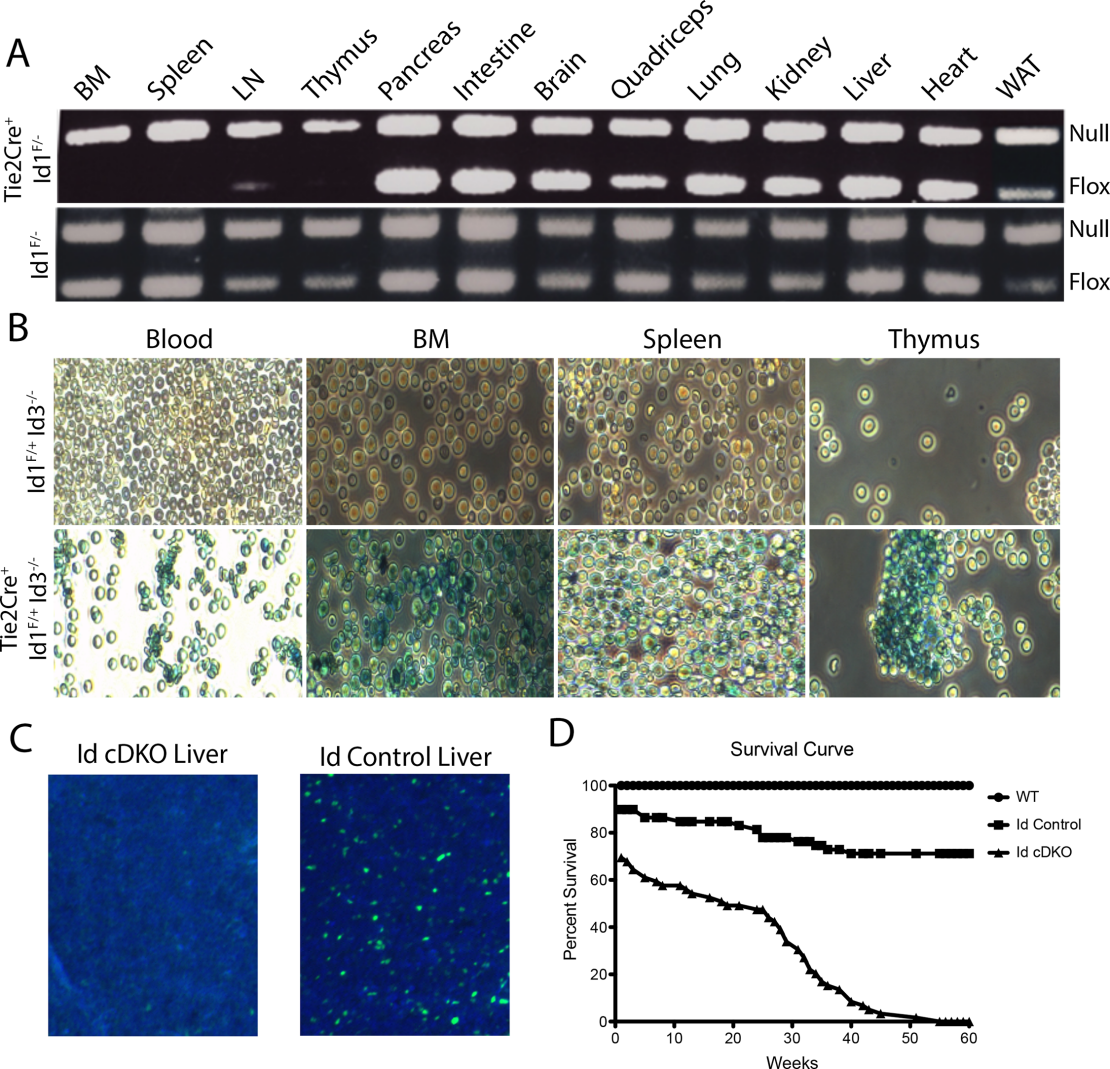
Tie2Cre-mediated Id1 deletion and Id3 global ablation reduced the lifespan of mice. A. Genomic PCR detecting Id1 in various tissues of 5–6 month old Id1 flox/ Id1 null mice with Tie2Cre (Tie2Cre^+^Id1^F/-^) or without Tie2Cre (Id1^F/-^). The Id1 null allele served as an internal DNA loading control. Tie2Cre converted the Id1 floxed allele into a Id1 null allele in all hematopoietic tissues without affecting mesenchymal tissues or any other tissues. WAT, white adipose tissue; LN, lymph node; BM, bone marrow. B. Xgal staining to visualize completed flox deletion in all hematopoietic tissues of 5–6 month old Tie2Cre/Rosa26-LacZ reporter mice. The floxed Id1 intermediate without Tie2Cre served as a negative control. (Magnification: 100X) C. Representative images of Id1 immunostaining in the developing embryonic liver (E13.5). Id1 protein was not detected in Id cDKO mice as a result of complete flox deletion of the Id1 gene. (Magnification: 40X) D. Survival curve of Id cDKO mice (n = 59), Id control mice (n = 59) and WT mice (n = 22).

Using Tie2Cre-mediated recombination, Tie2Cre^+^Id1^F/-^Id3^-/-^ or Tie2Cre^+^Id1^F/F^Id3^-/-^ (Id cDKO) mice were generated (Fig 1). Id cDKO mice were compared to both control littermates Id1^F/-^Id3^-/-^ or Id1^F/F^Id3^-/-^ without Tie2Cre recombination (Id control), where Id3 was deleted but one or two floxed alleles of Id1 remained, and WT mice. The floxed allele was deleted by Tie2Cre-mediated recombination in all hematopoietic tissues, including the bone marrow, spleen, thymus and lymph node (Fig 1A). The flox deletion was virtually complete in each hematopoietic tissue in 5–6 month old Tie2Cre/ROSA26-LacZ Cre Id1^F/F^Id3^+/-^ reporter mice (Fig 1B). Id1 protein was not observed in the developing liver, a major site of embryonic hematopoiesis, at mid-gestation in Id cDKO embryos (Fig 1C). Together, these analyses confirm that recombination occurred effectively in the hematopoietic compartment beginning as early as mid-gestational development. More than half of these mice survived embryogenesis, but the adults were small compared to littermate controls and died prematurely with a small percentage surviving past one year (Fig 1D). All subsequent data and analysis was performed on adult Id cDKO mice ranging from 5–6 months of age except where noted in the figure legends.

### Id cDKO mice exhibit anemia

**Fig 2:**
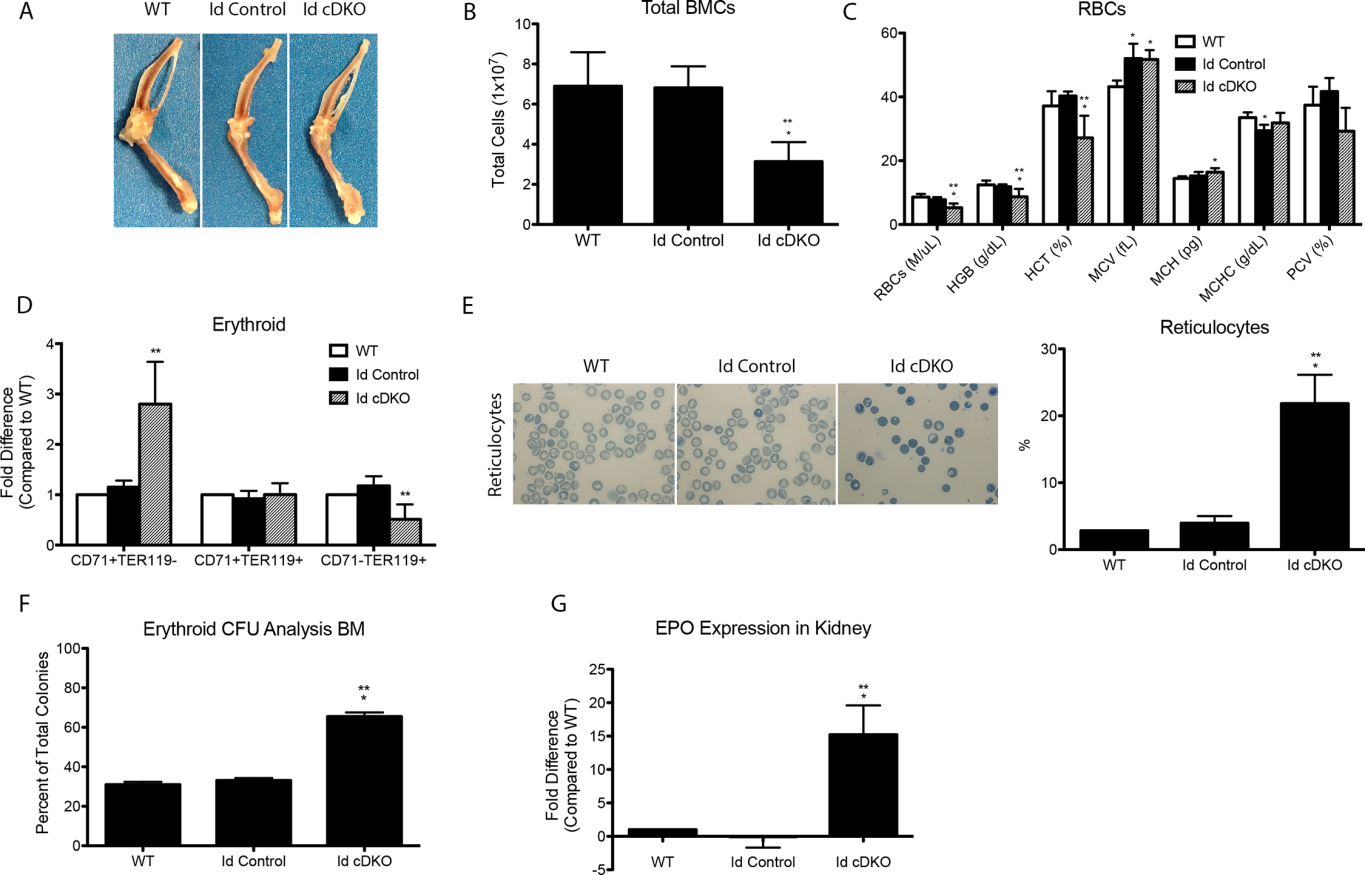
Id cDKO mice exhibit increased erythropoietin expression in kidneys, abnormal bone structure, and dysregulated bone marrow cells. 5–6 month old WT, Id control, and Id cDKO mice were analyzed unless otherwise noted. A. Representative images of femur and tibia/fibulas. B. Total number of bone marrow cells from femur and tibia of WT (n = 5), Id control (n = 6) and Id cDKO (n = 6) mice. C. RBC parameters from CBC analysis. RBC = red blood cell count, HGB = hemoglobin, HCT = hematocrit, MCV = mean corpuscular volume, MCH = mean corpuscular hemoglobin, MCHC = mean corpuscular hemoglobin concentration, PCV = packed cell volume D. Flow cytometric analysis of erythroid cells from early (CD71^+^TER119^-^) to late (CD71^-^TER119^+^) stages of development expressed as fold difference in percent of erythroid cells in gated bone marrow cells relative to WT levels. WT (n = 3), Id control (n = 4), Id cDKO (n = 4). E. Representative reticulocyte images with quantification (%) F. Colony forming unit assay. Percentage of erythroid colonies formed from different groups. WT (n = 3), Id control (n = 3), Id cDKO (n = 3). G. Erythropoietin (EPO) mRNA expression in kidneys of WT (n = 5), Id control (n = 5) and Id cDKO (n = 5) mice. *: p<0.05 compared to WT; **: p<0.05 compared to Id Control.

The bones of Id cDKOs appeared pale (Fig 2A), reflecting changes in bone marrow composition. Bone marrow cell quantification revealed that the total number of bone marrow nucleated cells (BMCs) was reduced by 55% in Id cDKO mice compared to Id control and WT mice (Fig 2B). Complete blood count (CBC) analysis of Id cDKO blood showed several erythrocyte abnormalities. The red blood cell (RBC) counts, hematocrit (HCT), and hemoglobin (HGB) concentrations in Id cDKOs were all significantly lower and out of normal range compared to those of Id controls and WTs (Fig 2C). Accordingly, the mean corpuscular volume (MCV) and mean corpuscular HGB content (MCH) of erythrocytes in Id cDKOs were significantly higher (but within normal range) than those of WTs, but not significantly different than those of Id controls. Packed cell volume (PCV) in Id cDKOs was significantly lower than that of both Id controls and WTs. Peripheral blood smears revealed an increase in number of reticulocytes in circulation (Fig 2E, left). The percentage of reticulocytes was significantly higher in Id cDKOs than both Id controls and WTs (Fig 2E, right). After adjusting for the drop in hematocrit, the reticulocyte index was found to be 15.9%, suggesting loss of red blood cells with an increased compensatory production of reticulocytes. Analysis of enhanced erythropoietin (*EPO*) expression in the kidneys of Id cDKOs showed a significant upregulation relative to WT and Id control levels (Fig 2G). Analysis of myeloid and lymphoid lineages from bone marrow revealed no significant change in Gr1+CD11b+ cells and a significant decrease in B220+ cells respectively (S1 Fig). Studies further investigated whether changes in the hematopoietic stem cell lineage (Lin- Sca1+ cKit+) could account for the observed erythroid deficiencies. Flow cytometry results showed no significant differences in percentage of HSCs between WT and Id cDKO mice at 6 months (S2 Fig).

### Id cDKO mice exhibit erythroid dysregulation

We next analyzed the percentage of erythroid cells in the bone marrow relative to the total number of cells (erythroid, CD71^+^ and TER119^+^). Immunophenotypic staging of erythroid cells in Id cDKO bone marrow revealed a shift towards earlier stages of erythoid development with a 2.9-fold (p<0.05) increase in CD71^+^TER119^-^ cells, no significant changes in CD71^+^TER119^+^ cells and a 1.6-fold (p<0.05) decrease in CD71^-^/TER119^+^ cells in Id cDKOs compared to WT controls (Fig 2D). No significant differences in the distribution of erythroid cells were seen between Id control and WT mice (Fig 2D). To assess progenitor function, we performed a colony forming unit (CFU) assay from bone marrow isolates and found a significant increase in erythroid colonies (65.5±1.18% in Id cDKOs vs. 30.96±0.81% in WTs) (Fig 2F).

### Id cDKO mice exhibit splenomegaly

**Fig 3:**
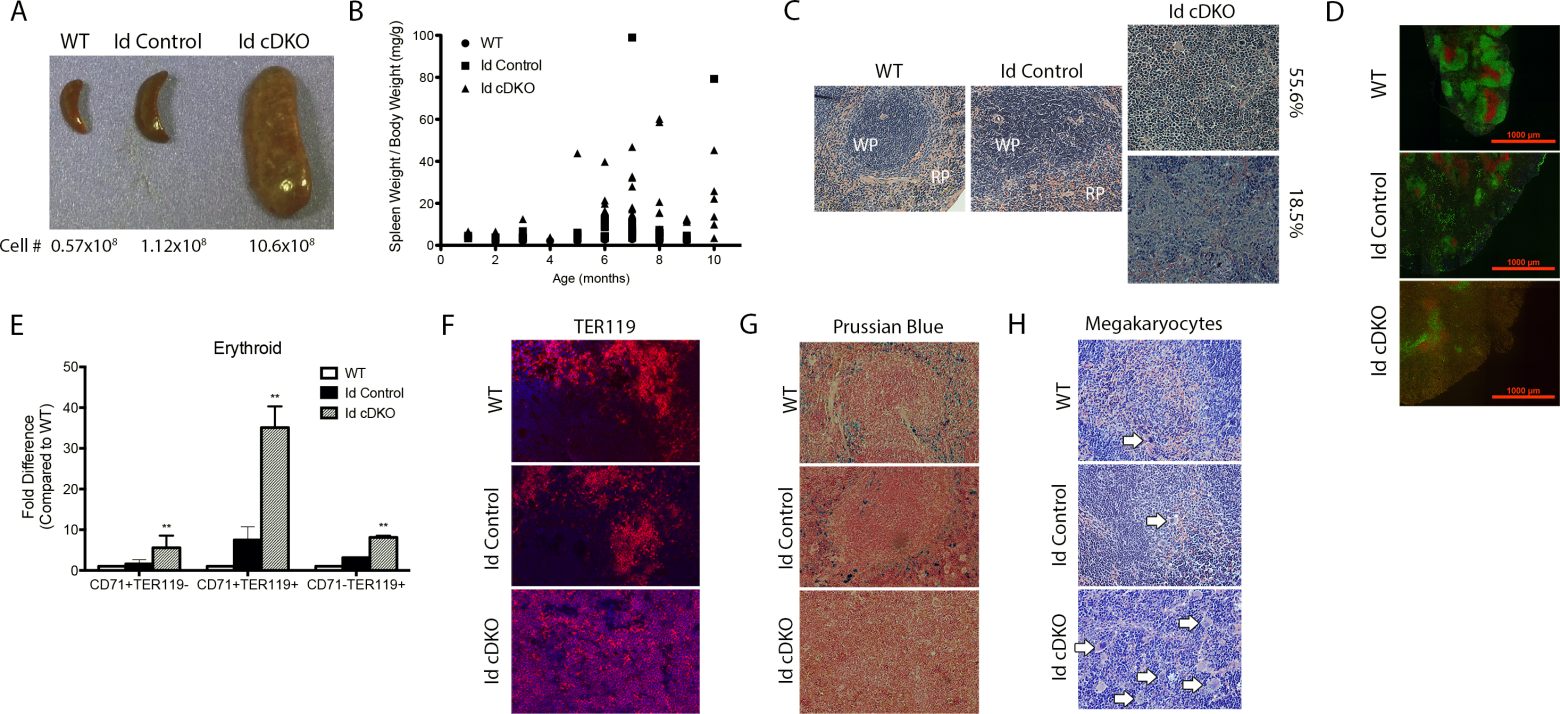
Splenomegaly and dysregulated splenocytes in Id cDKO mice. A. Gross morphology and total cell counts of representative spleens from 6 month old WT, Id control and Id cDKO mice. B. Spleen-to-body weight ratio (mg/g) represented in a scatter plot from WT (n = 26), Id control (n = 50) and Id cDKO (n = 49) mice at different ages. C. Representative H&E staining of spleens from WT, Id control and Id cDKO mice, WP = white pulp, RP = red pulp. (Magnification: 100X). D. Representative immunofluorescence staining of B220 (green) and CD3 (red) spleen sections from 6 month old WT, Id Control, and Id cDKO mice. E. Flow cytometric analysis of erythroid cells from early (CD71^+^TER119^-^) to late (CD71^-^TER119^+^) stages of development in 5–6 month old mice expressed as fold difference in percent of erythroid cells in gated splenocytes relative to WT levels. WT (n = 3), Id control (n = 4), Id cDKO (n = 4). F. Representative immunostain showing TER119^+^ erythroid cells in the spleen. (Magnification: 100X). G. Representative Prussian blue stains demonstrating distribution of released iron (blue color) in the spleen (Magnification: 40X). H. Representative H&E stains of spleens. Arrows indicate megakaryocytes. (Magnification: 100X). **: p<0.05 compared to WT and Id Control.

The size of the spleen in Id cDKO mice was 5- to 30-fold larger than that of WT mice with a total number of cells 5- to 30-fold higher (Fig 3A). The spleen weight/body weight ratio of Id cDKO mice was also found to be significantly higher than that of Id control and WT mice (Fig 3B). Histological analysis of the Id cDKO spleen revealed a markedly disorganized structure with effaced boundaries between red and white pulps. Out of 27 Id cDKO spleens, 15 displayed significant white pulp expansion (55.6%), 5 displayed significant red pulp expansion (18.5%) and 7 (25.9%) showed mixed features (Fig 3C and 3D). Immunophenotypic analysis revealed an 8.09-fold (p<0.05) increase in CD71^+^TER119^-^ cells, a 30-fold (p<0.05) increase in CD71^+^TER119^+^ and a 8.13-fold (p<0.05) increase in CD71^-^TER119^+^ cells in the Id cDKO spleen (Fig 3E). TER119^+^ immunostaining revealed diffuse distribution of erythroid cells in Id cDKO spleens (Fig 3F). Prussian blue stains of spleen sections revealed a marked decrease in iron deposits in Id cDKO spleens compared to both WT and Id control spleens (Fig 3G). Together, these results show that the Id cDKO spleen accumulates an increased number of early and intermediate-stage erythroid progenitors. The number of megakaryocytes in the spleen was also found to be increased in Id cDKO spleens, a sign of extramedullary hematopoiesis (Fig 3H). The percentage of Gr1^+^CD11b^+^ cells was significantly increased compared to WT levels while the percentage of B220^+^ cells were significantly decreased (S1 Fig). No significant changes were noted in the percentage of CD3^+^ cells within the spleen (S1 Fig). Overall, analysis of Id cDKO spleens reveal marked effacement of the splenic architecture and severe dysregulation in the development of the erythroid lineage.

### Defective hematopoietic microenvironments in Id cDKO mice

**Fig 4:**
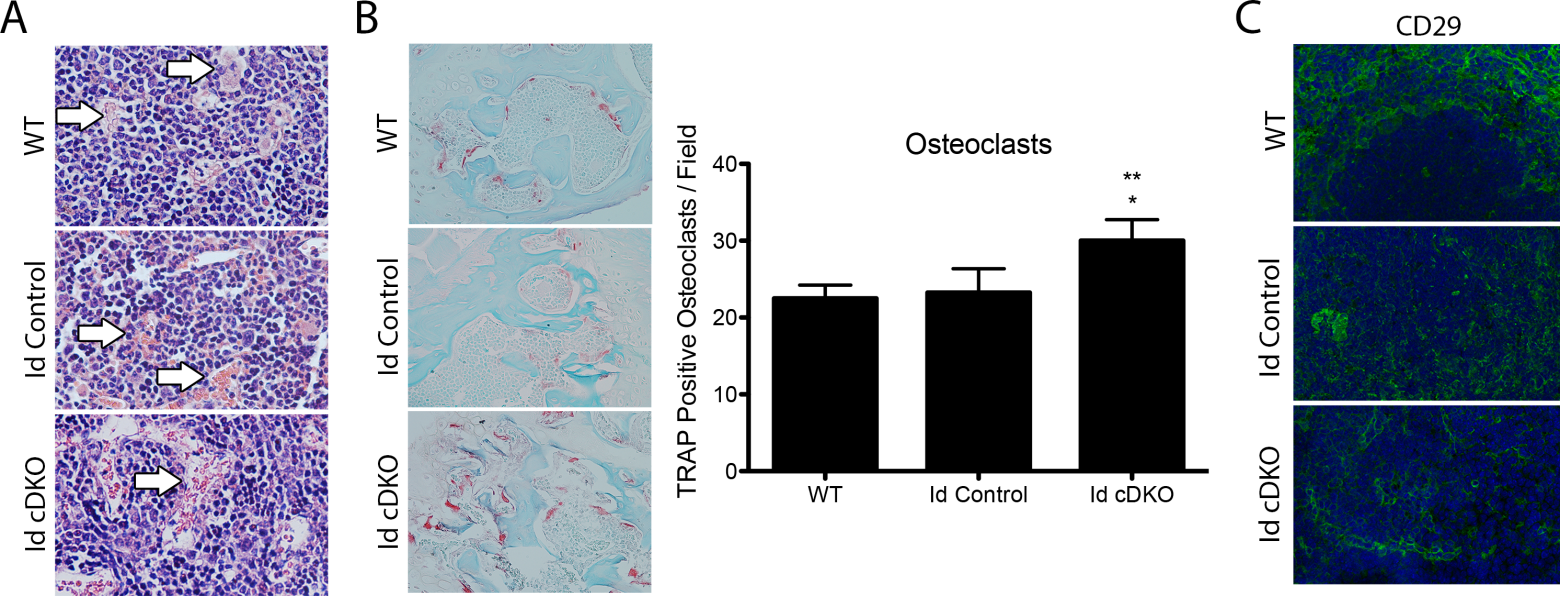
Defective hematopoietic microenvironments in Id cDKO mice. A. Representative H&E stain of longitudinal femur sections from 3 month old WT, Id control and Id cDKO mice. Arrows indicate vessels. (Magnification: 200X) B. TRAP-staining of osteoclasts (red) in femur sections (left) from 3-month-old WT, Id control and Id cDKO mice and quantification of number of TRAP^+^ osteoclasts per field (right). (Magnification: 200X) C. Representative immunofluorescence showing CD29^+^ endothelial cells in the spleen. (Magnification: 100X). *: p<0.05 compared to WT; **: p<0.05 compared to Id Control.

H&E stains of femurs showed abnormalities in bone architecture (Fig 4A). Since Tie2Cre targets the myeloid lineages, defects in bone structure could be attributed to alterations in osteoclast behavior and activity. Tartrate resistant alkaline phosphastase (TRAP) staining revealed an increased number of osteoclasts in Id cDKO bones compared to Id control and WT bones (Fig 4B). Morphologically, bone sections reveal a distended sinusoidal trabecular network (Fig 4A), consistent with increased osteoclastic activity. Tie2Cre also targets endothelial cells and CD29^+^ cell staining revealed a more disorganized and diffuse network of capillaries surrounding the white pulp of the Id cDKO spleen (Fig 4C).

### Extrinsic and intrinsic components of hematopoiesis in Id cDKO mice

**Fig 5:**
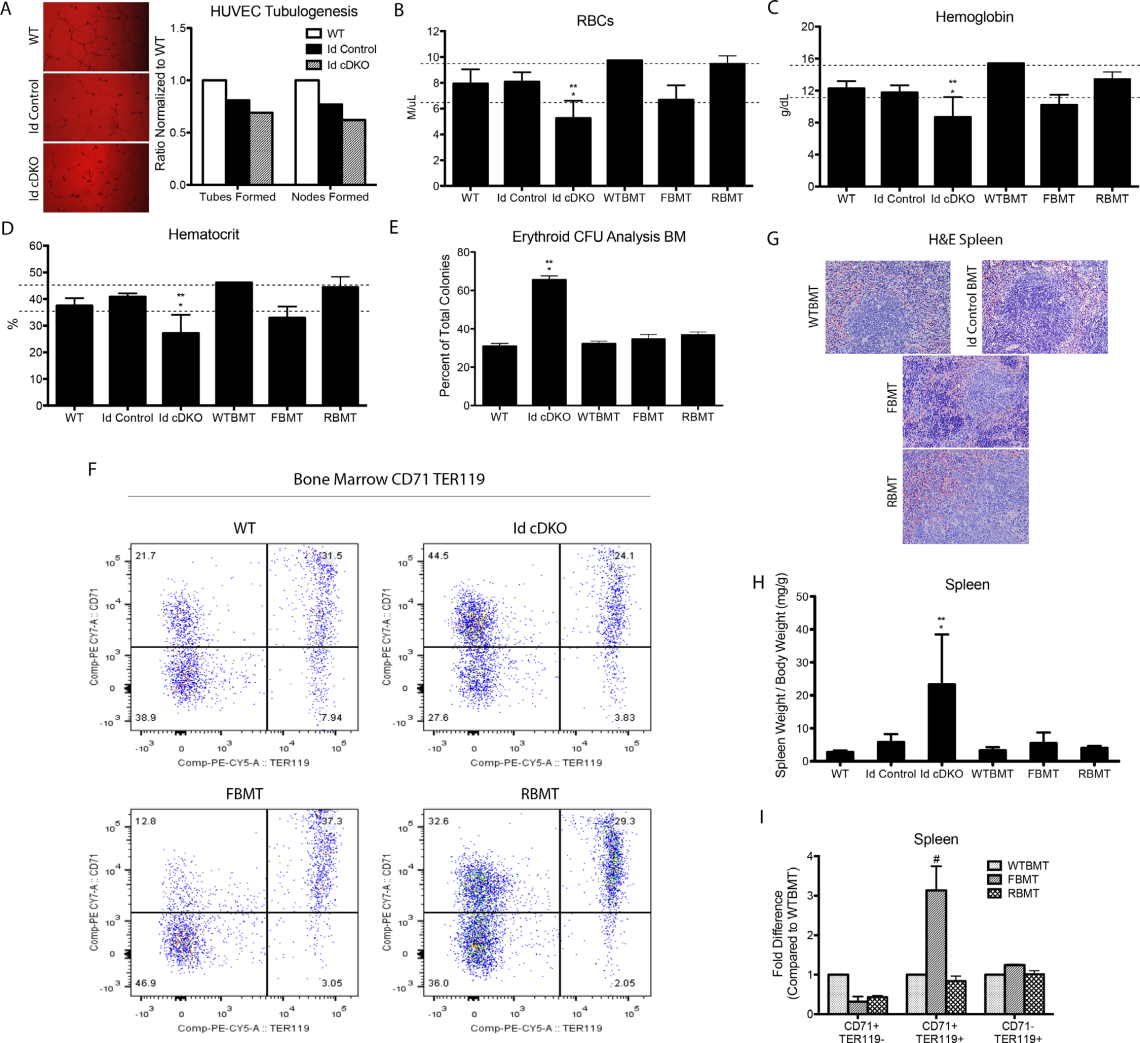
Intrinsic and extrinsic effects on anemia in Id cDKO mice. A. HUVEC in vitro tubulogenesis assay performed using WT, Id control and Id cDKO serum. Representative images (left, Magnification 200X) and quantification of differences in number of tubes and nodes formed (right). B-D. Comparison of red blood cell counts, hemoglobin and hematocrit between WT (n = 5), Id control (n = 5), Id cDKO (n = 5), WTBMT (n = 2), FBMT (n = 4) and RBMT (n = 4) mice. E. Colony forming unit assay. Percentage of erythroid colonies formed from different groups. Bone marrow cells isolated from 5–6 month old mice. WT (n = 3), Id cDKO (n = 3). WTBMT (n = 2), FBMT (n = 2), RBMT (n = 2). F. Representative flow cytometry maps of erythroid cells from early (CD71^+^TER119^-^) to late (CD71^-^TER119^+^) stages of development in 5–6 month old bone marrow transplanted mice. G. Representative H&E staining of spleens from bone marrow transplanted mice. WTBMT: WT donor/WT recipient; Id control BMT: Id control donor/WT recipient; FBMT: WT donor/Id cDKO recipient; RBMT: Id cDKO donor/WT recipient (Magnification: 100X)(for WT, Id control and Id cDKO, see Fig 3C). H. Ratio of spleen weight to body weight (mg/g) from 5–6 month old WT (n = 26), Id control (n = 50), Id cDKO (n = 49), WTBMT (n = 5), FBMT (n = 9), RBMT (n = 9). I. Flow cytometric analysis of erythroid cells in spleen from early (CD71^+^TER119^-^) to late (CD71^-^TER119^+^) stages of development. Fold difference in percent of erythroid cells in gated splenocytes relative to WTBMT levels. *: p<0.05 compared to WT; **: p<0.05 compared to Id Control.

To better understand whether this endothelial cell behavior had an extrinsic component in a system where both hematopoietic and endothelial compartments are affected, WT human umbilical vein endothelial cells (HUVEC) were treated with serum from WT, Id control and Id cDKO mice. Findings revealed HUVECs displayed a number of defects in tube formation including a 30.7% decrease in number of tubes and a 38.4% decrease in number of nodes upon incubation with Id cDKO serum compared to treatment with WT serum (Fig 5A). Serum composition was analyzed using mass spectrometry. Several factors important for angiogenesis, including insulin-like growth factor binding protein 2 (IGFbp2) and thrombospondin 4 (Tbp4), were found upregulated in serum from Id cDKO mice compared to WT controls (S1 Table). These findings are consistent with prior studies showing dystregulation of thrombospondin 1 (TSP1) and insulin-like growth factor binding protein 3 (IGFbp3) in the hearts of 6 month-old Id cDKO mice [16].

Studies next sought to examine whether the hematopoietic defects observed in Id cDKO mice had extrinsic components like the Id-expressing (endothelial) component of hematopoietic niches. To address this question, we conducted a series of bone marrow transplantation experiments in lethally irradiated mice (Fig 5B–5H). No significant differences were observed in any of the observed parameters between WT mice transplanted with WT bone marrow (WTBMT) and WT mice (Fig 5B–5I).

First, GFP^+^ WT bone marrow cells were injected into Id cDKO mice (‘forward’ or FBMT). Full bone marrow reconstitution (>95%) occurred. RBC parameters of FBMT blood (RBCs, hemoglobin, hematocrit) were increased compared to Id cDKO mice, but remained lower than WTBMT levels (Fig 5B–5D). From the three RBC parameters assessed, the RBC count was the only parameter that fell within normal range. CFU analysis of FBMT bone marrow cells revealed a colony distribution similar to the WTBMT profile with BFU-E constituting approximately 34.75±2.31% of total colonies (Fig 5E). FACS analysis of erythroid lineages from bone marrow of FBMT mice revealed a similar distribution of erythroid populations compared to WT mice (Fig 5F).

The spleen size of FBMT mice was observed to reach Id control levels (Fig 5H). Histologically, FBMT spleens showed improvements in splenic architecture with partial restoration of red pulp and white pulp integrity (Fig 5G). However, structural abnormalities remained evident. FACS analysis of FBMT splenocytes revealed a 5.17-fold increase in CD71^+^TER119^+^ cells and 3.44-fold decrease in CD71^+^TER119^-^ cells relative to WTBMT levels (Fig 5I). No significant fold changes in CD71^-^TER119^+^ cells were observed.

We also conducted a reverse bone marrow transplant (‘reverse’ or RBMT), which involved injection of Id cDKO bone marrow cells into lethally irradiated GFP^+^ WT mice. Full bone marrow reconstitution (>95%) occurred. RBC parameters of RBMT blood (RBCs, hemoglobin, hematocrit) were within normal range (Fig 5B–5D). CFU analysis of RBMT bone marrow cells revealed a restoration of erythroid colonies to WTBMT levels at 36.90±1.41% (Fig 5E). FACS analysis of erythroid lineages from bone marrow of RBMT mice revealed a similar distribution of erythroid populations in RBMT mice compared to Id cDKO mice, with a similar increase in CD71^+^TER119^-^ (early erythroid) cell population (Fig 5F).

The spleen weight-to-body weight ratio of RBMT mice reached Id control levels (Fig 5H). Histologically, RBMT spleens still exhibit intact red and white pulps but the boundaries were less defined and structural changes were apparent compared to both WTBMT and Id control BMT spleens (Fig 5G). No significant changes in histology were noted between WTBMT and WT spleens (Figs 3C and 5G). FACS analysis of RBMT splenocytes showed an erythroid distribution similar to the WTBMT profile except for a 2.5-fold reduction in CD71^+^TER119^-^ cells (Fig 5I).

### Disruption of the hematopoietic transcriptional network in Id cDKO mice

**Fig 6:**
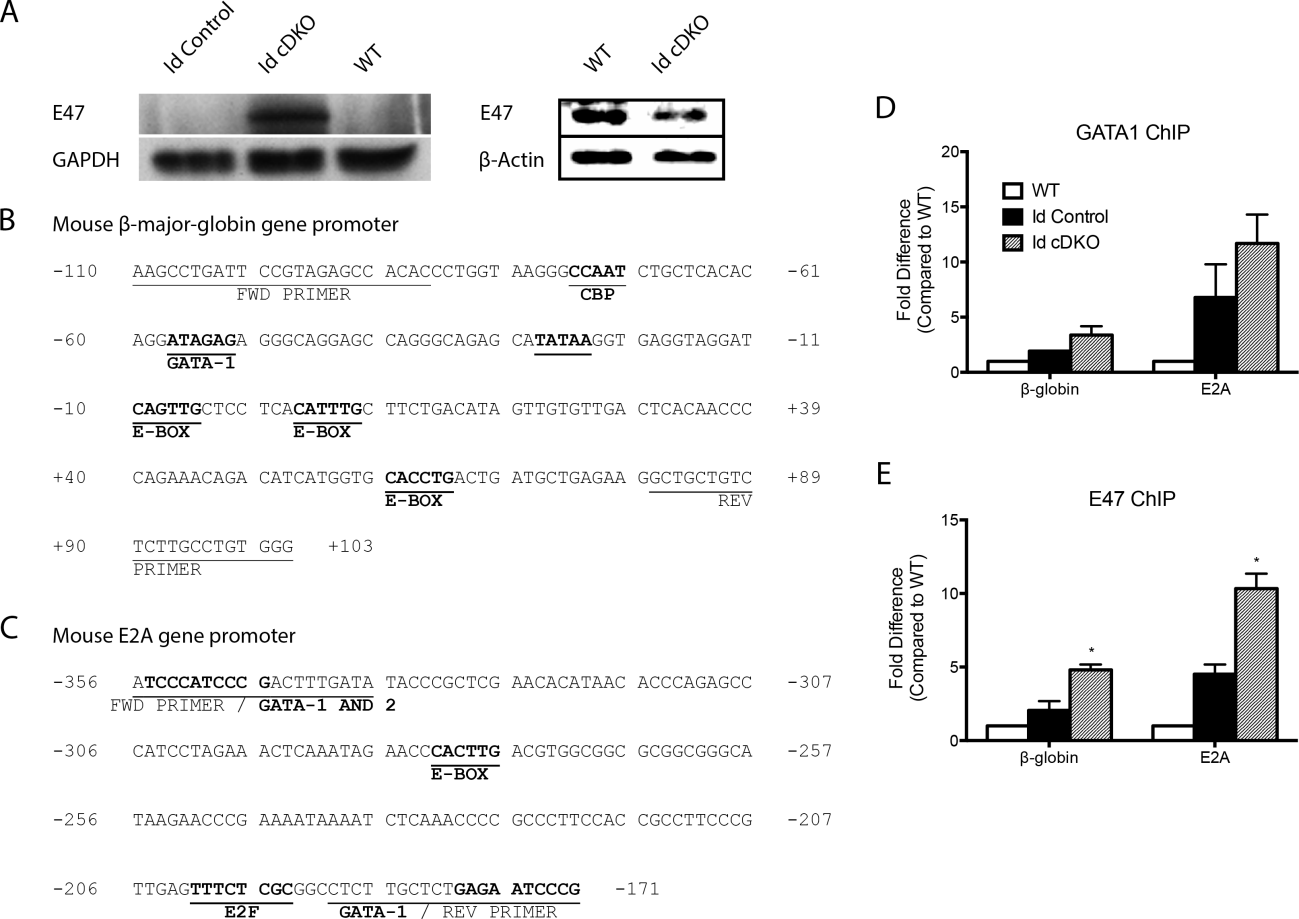
Bone marrow GATA1 and E47 display changes in occupancy to promoter regions of β-globin and E2A. A. Western blot measurement of E47 (product of E2A) from whole bone marrow cells (left, top row) and erythroid cells (CD71^+^TER119^+^, right, top row) in WT, Id control, and Id cDKO mice at 6 months of age. Protein levels were normalized to GAPDH or β-actin levels (bottom row). B. Schematic of mouse β-globin gene promoter region with location of PCR primers and known E-box and GATA binding sites. Protein binding sites and conserved consensus sequences are underlined. C. Schematic of mouse E2A gene promoter region with location of PCR primers and location of known E-box and GATA binding sites. D. ChIP measurement of fold change in occupancy of GATA1 to the promoter region of β-globin and E2A. E. ChIP measurement of fold change in occupancy of E47 to the promoter region of β-globin and E2A. *: p<0.05 compared to WT.

Loss of Id led to dysregulation of the hematopoietic transcriptional machinery. Proteomic analysis of Id cDKO relative to WT bone marrow revealed significant changes in markers associated with hematopoietic system development and function, immune trafficking, cell death, cell growth and proliferation (S2 Table). In both the bone marrow and spleen of Id cDKO mice several important hematopoietic transcription factors were upregulated (*E2A*, *GATA1*, *Scl*, *Zfpm1*, *Lmo2*) while Pu.1 was downregulated (Table 1). Fold changes in the expression of these genes in the spleen were more prominent than in the bone marrow (Table 1). Other factors like *GATA2* were minimally dysregulated (Table 1). WB analysis revealed an increase in E47 protein levels within Id cDKO bone marrow cells (Fig 6A). However, E47 levels in erythroid lineages (CD71^+^TER119^+^ cells) were decreased (Fig 6B). Chromatin immunoprecipitation assays using primers specific to the upstream promoter regions of *β-globin* and E2A revealed increased occupancy of both GATA1 and E47 at the promoter region of *β-globin* and E2A (Fig 6B–6E) [41,42].

**Table 1:** Dysregulation of various members of the hematopoietic transcriptional network. The values in the Id control and Id cDKO column reflect fold differences in mRNA expression with respect to WT levels. Values in the FBMT and RBMT columns reflect fold differences in mRNA expression with respect to WTBMT levels.

	Id Control	Id cDKO	FBMT	RBMT
**BM**				
GATA1	2.40±0.36 *	10.52±2.28 *	-0.31±1.14	1.03±1.11
GATA2	1.26±0.35	1.88±0.46	0.28±1.32	-0.66±1.49
Lmo2	1.14±0.09	3.20±0.40 *	-2.54±1.70	1.28±1.72
Zfpm1	-1.49±0.20 *	3.24±0.34 *	4.60±2.32	3.33±3.40
Pu1	-0.94±1.59	-6.33±2.60 *	0.42±1.40	0.48±1.84
Scl	1.68±0.43	6.01±0.75 *	1.78±2.03	6.25±4.47
E2A	7.40±2.57 *	14.31±9.12	0.64±0.46	1.85±0.53
β-globin	-2.01±0.33 *	-7.79±0.46 *	-0.78±0.03 #	-10.22±1.28 #
**Spleen**				
GATA1	4.10±0.69 *	59.95±2.36 *	2.51±1.75	0.15±0.83
GATA2	3.53±0.23 *	3.29±0.29 *	-2.24±1.50	-0.49±1.12
Lmo2	1.50±0.66	9.35±1.30 *	-0.16±0.81	-0.45±1.04
Zfpm1	2.97±0.02*	17.95±1.29 *	10.93±9.63	1.36±3.44
Pu1	-3.93±0.95 *	-3.32±0.49 *	1.40±1.04	6.19±3.77
Scl	2.92±1.03	287.48±82.16 *	28.15±14.22	13.02±7.30
E2A	24.48±0.21 *	108.51±7.44 *	1.42±1.25	1.62±1.56
β-globin	-26.45±7.23 *	-78.89±14.05 *	1.25±0.91	4.64±3.98

*: p< 0.05 compared to WT levels.

#: p<0.05 compared to WTBMT levels.

Bone marrow transplantation studies reveal normalization of several members of the hematopoietic transcriptional machinery including E2A, *GATA1*, *Lmo2*, *Zfpm1*, *Pu*.*1* and *Scl* in the bone marrow and spleen of FBMT and RBMT mice (Table 1). One key target gene for erythropoiesis, *β-globin* (a mature erythroid marker), was downregulated in the bone marrow and spleen (-7.8-fold and -78.9-fold respectively, relative to WT) (Table 1). This level was normalized in FBMTs but remained significant in the bone marrow of RBMT mice (Table 1). Taken together, these results suggest that loss of Id compensation leads to a severe dysregulation of a downstream transcriptional network, affecting master regulators of hematopoiesis with strong intrinsic and extrinsic regulatory circuitry.

## Discussion

Disruption of Id1 and Id3 genes in Id cDKOs led to the development of multiple hematopoietic disturbances including anemia. Such an effect can be attributed to prior evidence that Id1 and Id3 are expressed in similar overlapping patterns within the embryo during development with expression persisting into adulthood in hematopoietic and endothelial cells [43]. Although Id2 and Id4 are known to exist, Id4 is not expressed in hematopoietic cells and Id2’s role in hematopoietic differentiation has not been shown to be sufficient to compensate for the combined loss of Id1 and Id3 [43,44]. This was primarily associated with an increased percentage of immature nucleated red blood cells in the peripheral blood, decreased bone marrow cellularity and increased numbers of bone marrow erythroid precursors. While the anemia appears to trigger a robust bone marrow response to produce more erythroid cells, as evidenced by the marked reticulocytosis, the bone marrow output is compromised and erythroid development is impaired. Despite the presence of this robust bone marrow response, Id cDKO mice develop anemia with overall ineffective erythropoiesis characterized by a large proportion of immature precursors. While our study did not identify differences at the HSC level at 6 months of age, further studies may help elucidate whether HSC behavior (i.e., differentiation, self-renewal, quiescence) is altered. Moreover, whether the observed anemia responds to blood transfusion or splenectomy remains to be determined. This erythroid defect has not been reported in prior models of Id1 or Id3 ablation [29,30,34,35].

These erythroid changes appear less severe in both transplantation groups. In FBMTs, decreases in RBC parameters despite the presence of intact Id signals in the bone marrow cells might reflect a strong extrinsic effect from an Id-deficient niche (endothelial). These findings support a previous study in which the Id1 KO environment was shown to be defective with significant extrinsic influences in hematopoiesis [29]. While the resemblance of the bone marrow erythroid profile of FBMT mice to the WT profile lends support to the maintenance of intrinsic regulatory mechanisms in donor bone marrow cells, the slight increase in spleen size raises the possibility that the observed mild anemia may be secondary to hypersplenism, as the recipient spleen is still architecturally defective. The extent to which bone marrow derived endothelial cells may contribute to the nascent vasculature and remodeling of the recipient spleen remains to be determined. Furthermore, the anemia in FBMT mice is not as severe as the fulminant pathology observed in Id cDKO mice and may not be sufficient to trigger compensatory mechanisms (i.e., extramedullary hematopoiesis).

Analysis of RBMTs revealed that although the donor bone marrow cells were genotypically Id-deficient, these mice did not develop anemia despite data demonstrating an increased presence of early immature erythroid cell population. While it is possible that hematopoietic microenvironments may play an important role in regulating hematopoiesis, the increased presence of immature erythroid cells in RBMT mice suggests that the grafted bone marrow cells are still intrinsically defective. The key to understanding this discrepancy might lie in the significant remodeling observed in RBMT spleens, which may reflect increased extramedullary hematopoiesis that compensates for the intrinsically defective bone marrow derived erythroid cells. It is also possible that RBMT mice may develop anemia at later stages of life, a question that remains to be investigated in more long-term studies.

The Id cDKO spleen enlarged on average to levels greater than typically seen in either Id1 KO or Id3 KO mice. Our findings reveal that Id3 KO mice do not develop anemia, although a small fraction progress to develop splenomegaly by 10 months. The Id3 KO mice that develop splenomegaly show lymphomatous changes consistent with a previous report [35]. Additionally, Id1 KO mice were found to display no anemia or splenomegaly (data not shown). Id cDKO spleens appear to develop fulminant pathological changes by 6 months of age. Diffuse distribution of immature erythroid cells in Id cDKO spleens seems to reflect loss of integrity of the red pulp. Elevated levels of splenic megakaryocytes raise the possibility of extramedullary hematopoietic compensation. Interestingly, Id cDKOs have not been observed to develop anemia or splenomegaly at 1 month of age (data not shown), yet by 6 months of age, both phenotypes are apparent. An association between the two phenotypes is present but whether the splenomegaly occurs as a compensatory response to the anemia or as the cause of the anemia remains to be determined. Elevated *EPO* production in Id cDKO kidneys may provide a stimulus for extramedullary hematopoiesis. Increased *EPO* levels trigger the JAK2/STAT5 signaling pathway and upregulate Id1 expression, which is believed to promote erythroblast survival and decrease erythroid apoptosis [45].

Decreased levels of both iron deposits and iron-positive macrophages in the spleen raise the possibility that Id cDKO splenic macrophages may have an intrinsic defect in their capacity to sequester abnormal or immature RBCs from circulation, which may impair recovery of iron to the hematopoietic system so that newly formed erythroid precursors fail to incorporate iron. Significant downregulation of beta-globin coupled with preliminary findings of dysregulation of genes involved in iron homeostasis suggest defects in hemoglobin complex formation and iron handling.

It is noteworthy to mention the compromised endothelial network of Id cDKO bones and spleens. Since Id cDKO serum has a negative impact on the in vitro endothelial tube growth capacity of WT endothelial cells, it is possible that dysregulated factors from the Id cDKO serum may contribute to the endothelial phenotype. Our study demonstrated dysregulation of important secreted, angiogenic factors within the serum of Id cDKO that may contribute in part to the endothelial phenotype observed in these mice. While bone marrow cells may be influenced by extrinsic signals derived from the hematopoietic niches, hematopoietic microenvironments may also undergo remodeling following repopulation and perhaps, in response to signals secreted by bone marrow cells. The splenic architecture of FBMT and RBMT mice appeared less severely disrupted compared to the fulminant Id cDKO pathology raising the question of how and to what extent grafted cells (i.e., hematopoietic cells, bone marrow derived endothelial cells) contribute to the nascent vasculature and to the remodeling process within the spleen.

E2A, the product of which is an Id partner and target for Id sequestration [1], was enhanced in the Id cDKO bone marrow and spleen. E47 protein levels were markedly elevated in Id cDKO bone marrow. This combined effect of loss of Id compensation, which liberates E proteins, and transcriptional enhancement of E2A may have led to a robust amplification cascade of dysregulation of the Id/E pathway that may have included tissue-specific bHLH proteins, like Scl [46,47]. However, E47 protein levels in an enriched fraction of erythroid cells (CD71^+^TER119^+^) were decreased compared to WT levels, suggesting that E47 enhancement may be found in other lineages. A previous study investigating Id1/E2A double null mice has not yet reported erythroid disturbances [48]. While most of the master regulators of hematopoiesis that we analyzed were upregulated, like E2A, GATA1 and Scl, other factors were down-regulated, like PU.1 in the spleen and bone marrow. The amplification of transcription of these genes in the spleen compared to the bone marrow specifically within the Id cDKO supports the idea that this organ is an important site of active hematopoiesis and hematopoietic dysregulation in these mice. ChIP analysis of Id cDKO bone marrow cells demonstrate increased occupancy of both E47 and GATA1 at the promoter region of *β-globin*. Increased occupancy of E47 at the *β-globin* gene promoter is consistent with the notion that Id deletion leads to increased E2A DNA-binding activity. The increased occupancy of E2A may also reflect the increased percentage of developing erythrocytes, which possess higher E2A activity. It is known that E2A binding is associated with activation of globin genes [49]. Thus, the decreased globin gene expression we observe may not be entirely a consequence of a direct effect, and opposing indirect effects may come into play.

ChIP analysis also revealed increased occupancy of E47 and GATA1 at the promoter region of *E2A*, raising the possibility of an autoregulatory loop. Most of the transcriptional factors that did respond within the transplantation studies are members of the master hematopoietic transcriptional machinery (e.g., *GATA1*, *Lmo2*, *Zpfm1*, *Scl*). Normalization of these factors in transplantation studies suggest not only that this transcriptional network is tightly regulated in bone marrow cells, allowing for proper development in the presence of an Id deficient environment, but also that homeostasis of gene expression in this transcriptional network can be partially restored via extrinsic influences from the WT environment. Minimal alterations in these factors in transplanted mice may also reflect differences in latency of phenotypic manifestation, as more robust gene expression changes may be observed immediately after bone marrow transplantation. Likewise, it cannot be ruled out that the minimal alterations observed in RBMT mice obey to the fact that developmental hematopoiesis proceeds normally, since transplantation takes place after birth.

Together these findings provide a basis for understanding the impact of loss of Id compensation on the hematopoietic transcriptional machinery. The findings link reciprocal interactions between two developmentally related tissues, the endothelium and hematopoietic cells. Thus, we have unveiled a novel role for Id in erythropoiesis using a recombination driver that targets the tissue of study and its niche. Ultimately, understanding how modulation of Id function affects downstream hematopoietic regulators will have significant implications for achieving greater transcriptional control over hematopoietic specification.

## Supporting Information

S1 Fig Alterations in Myeloid and Lymphoid flow cytometric profile in Id cDKO mice. A) Percentage of gated Gr1^+^CD11b^+^ cells within the bone marrow. B) Percentage of gated B220^+^ cells within the bone marrow. C) Percentage of gated Gr1^+^CD11b^+^ cells within the spleen. D) Percentage of gated B220^+^ cells within the spleen. E) Percentage of gated CD3^+^ cells within the spleen. *: p<0.05 with respect to WT levels. **: p<0.05 compared to Id control levels.
(TIF)

S2 Fig Flow cytometric analysis of hematopoietic stem cell populations (Lin^-^Sca1^+^cKit^+^) in Id cDKO mice. Analysis was performed on n = 2 WT mice, n = 2 Id control mice, and n = 3 Id cDKO mice at 6 months of age.
(TIF)

S1 Table Proteomic analysis of Id cDKO serum. Analysis was performed on n = 2 WT mice and n = 2 Id cDKO mice. Cut-off: T-test p = 0.05.
(PDF)

S2 Table Pathway analysis of complex omics data for Id cDKO bone marrow cells. Analysis was performed on n = 2 WT mice and n = 3 Id cDKO mice.
(PDF)
