# Supplementary figures and images for: Combined Id1 and Id3 Deletion Leads to Severe Erythropoietic Disturbances
Source: PLoS One. 2016 Apr 29;11(4):e0154480. doi: 10.1371/journal.pone.0154480 (PMC4851361; doi:10.1371/journal.pone.0154480)

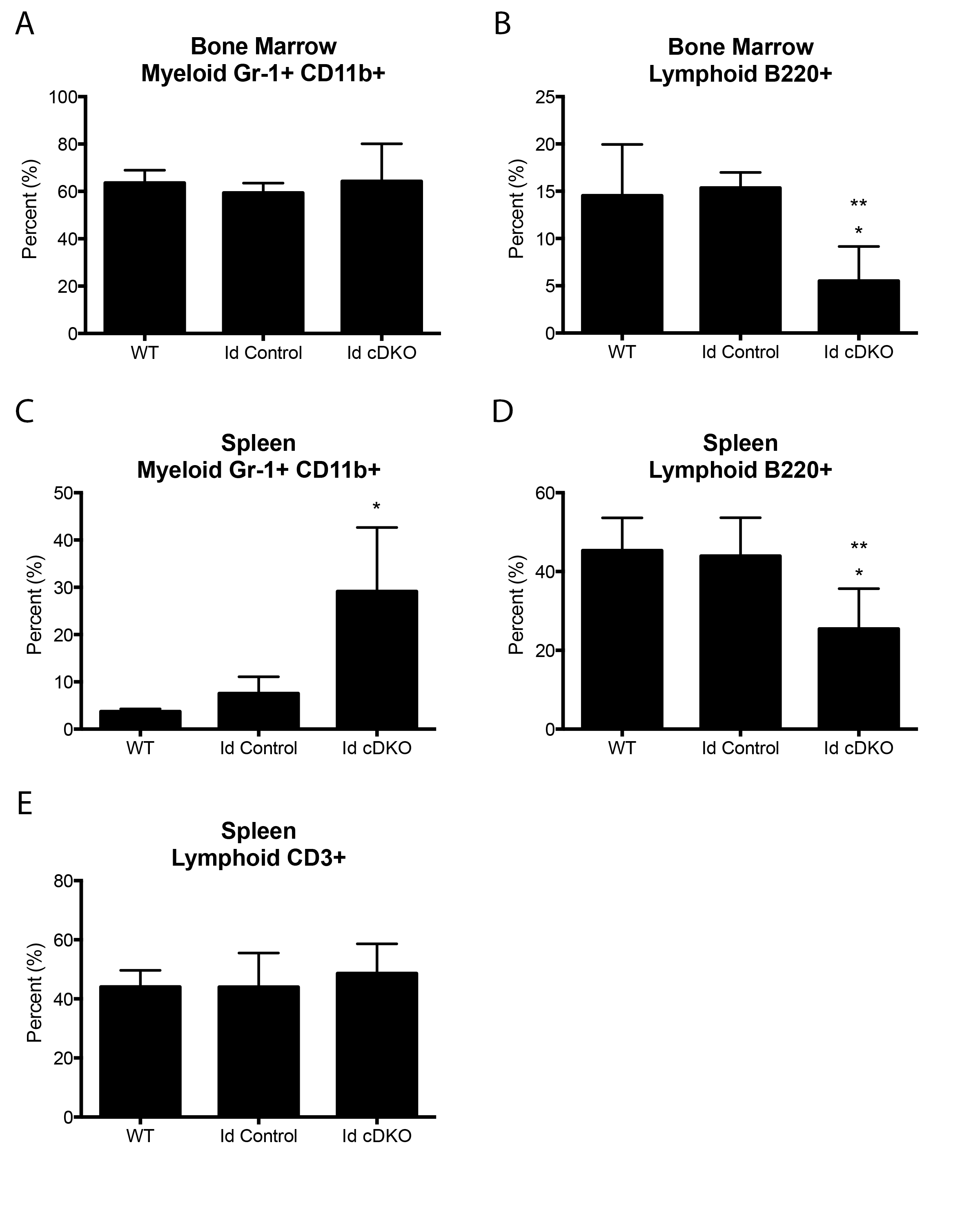

Supplement: S1 Fig — A) Percentage of gated Gr1+CD11b+ cells within the bone marrow. B) Percentage of gated B220+ cells within the bone marrow. C) Percentage of gated Gr1+CD11b+ cells within the spleen. D) Percentage of gated B220+ cells within the spleen. E) Percentage of gated CD3+ cells within the spleen. *: p<0.05 with respect to WT levels. **: p<0.05 compared to Id control levels. (TIF) [file pone.0154480.s001.tif]

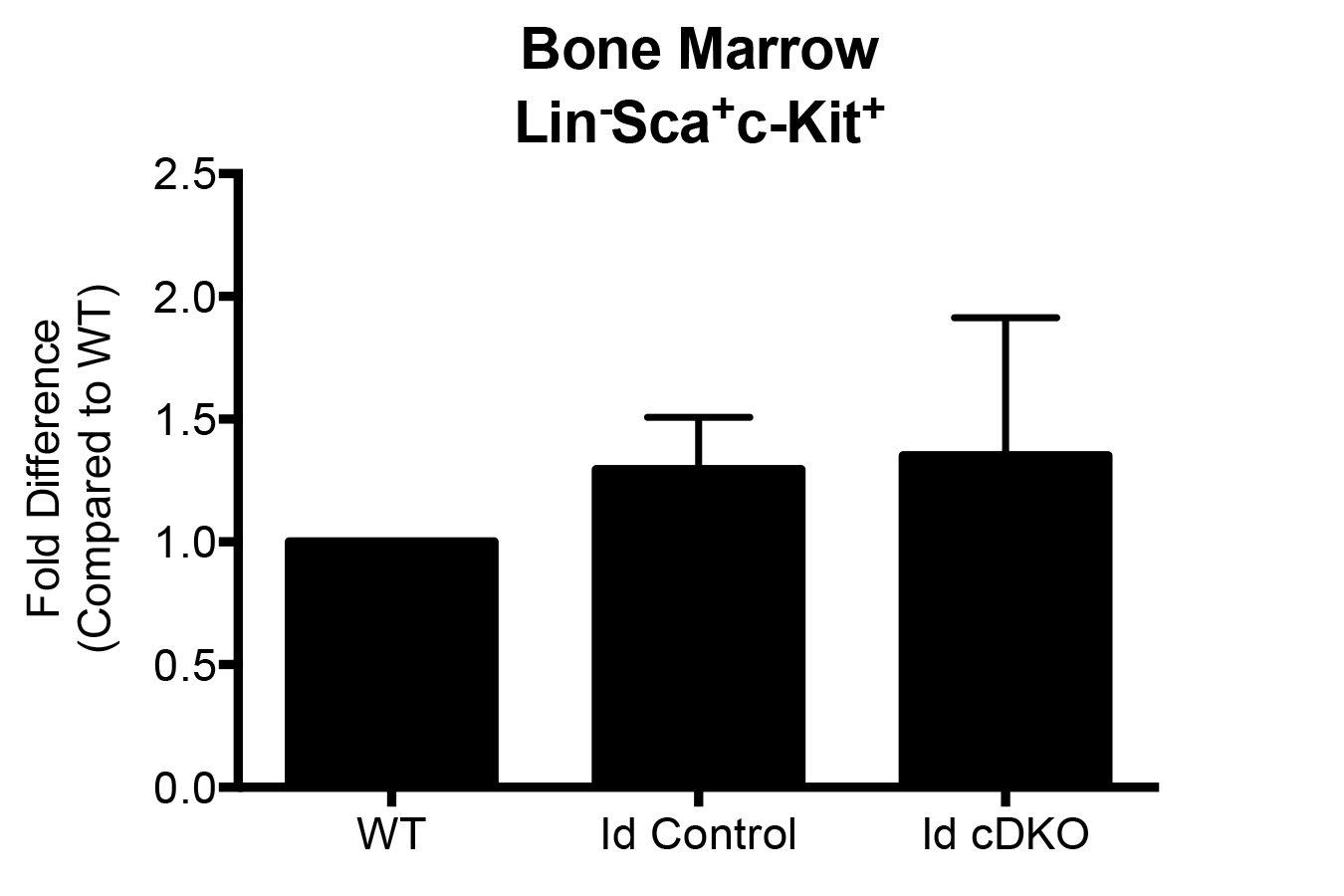

Supplement: S2 Fig — Analysis was performed on n = 2 WT mice, n = 2 Id control mice, and n = 3 Id cDKO mice at 6 months of age. (TIF) [file pone.0154480.s002.tif]
