# Supplementary material for: Combined Id1 and Id3 Deletion Leads to Severe Erythropoietic Disturbances
Source: PLoS One. 2016 Apr 29;11(4):e0154480. doi: 10.1371/journal.pone.0154480 (PMC4851361; doi:10.1371/journal.pone.0154480)
Supplement: S1 Table — Analysis was performed on n = 2 WT mice and n = 2 Id cDKO mice. Cut-off: T-test p = 0.05. (PDF) [file pone.0154480.s003.pdf]

S1 Table: List of identified proteins and their relative quantitation based on Spectra counting (sorted by T-test)

Identified Proteins (580)	Accession Number	Molecular Weight	Spectra counts (WT1)	Spectra counts (WT2)	Spectra counts (cKO63)	Spectra counts (cKO67)	Ratio (cKO/WT)	T-test
Glutamyl aminopeptidase OS=Mus musculus GN=Enpep I	AMPE_MOUSE	108 kDa	1	1	0	0	0.5	0.00
Neural cell adhesion molecule L1 OS=Mus musculus GN=	L1CAM_MOUSE	141 kDa	1	1	0	0	0.5	0.00
60S ribosomal protein L22 OS=Mus musculus GN=Rpl22 F	RL22_MOUSE	15 kDa	1	1	0	0	0.5	0.00
Rho GDP-dissociation inhibitor 1 OS=Mus musculus GN=/	GDIR1_MOUSE	23 kDa	1	1	0	0	0.5	0.00
Bisphosphoglycerate mutase OS=Mus musculus GN=Bpgr	PMGE_MOUSE	30 kDa	1	1	0	0	0.5	0.00
Sorbitol dehydrogenase OS=Mus musculus GN=Sord PE=1	DHSO_MOUSE	38 kDa	1	1	0	0	0.5	0.00
Pleckstrin OS=Mus musculus GN=Plek PE=1 SV=1	PLEK_MOUSE	40 kDa	1	1	0	0	0.5	0.00
Indian hedgehog protein OS=Mus musculus GN=Ihh PE=1	IHH_MOUSE	45 kDa	1	1	0	0	0.5	0.00
Tubulin beta-1 chain OS=Mus musculus GN=Tubb1 PE=1	TBB1_MOUSE	50 kDa	1	1	0	0	0.5	0.00
Corepressor interacting with RBPJ 1 OS=Mus musculus G	CIR1_MOUSE	52 kDa	1	1	0	0	0.5	0.00
Uromodulin OS=Mus musculus GN=Umod PE=1 SV=1	UROM_MOUSE	71 kDa	1	1	0	0	0.5	0.00
Leucine-rich repeat neuronal protein 4 OS=Mus musculus	LRRN4_MOUSE	80 kDa	1	1	0	0	0.5	0.00
Keratin, type II cuticular Hb5 OS=Mus musculus GN=Krt8!	KRT85_MOUSE	56 kDa	4	4	0	0	0.2	0.00
Dipeptidyl peptidase 4 OS=Mus musculus GN=Dpp4 PE=1	DPP4_MOUSE	87 kDa	2	2	0	0	0.3	0.00
Ig kappa chain V-II region MOPC 167 OS=Mus musculus P	KV2A1_MOUSE	12 kDa	0	0	1	1	2.0	0.00
Ig lambda-1 chain V region S178 OS=Mus musculus PE=1	LV1C_MOUSE	12 kDa	0	0	1	1	2.0	0.00
Ig heavy chain V region OS=Mus musculus PE=1 SV=1	HVM00_MOUSE	13 kDa	0	0	1	1	2.0	0.00
Troponin T, cardiac muscle OS=Mus musculus GN=Tnnt2	TNNT2_MOUSE	36 kDa	0	0	1	1	2.0	0.00
Dentin sialophosphoprotein OS=Mus musculus GN=Dspp	DSPP_MOUSE	94 kDa	0	0	1	1	2.0	0.00
Apolipoprotein C-IV OS=Mus musculus GN=Apoc4 PE=1 S	APOC4_MOUSE	14 kDa	2	2	1	1	0.7	0.00
Insulin-like growth factor-binding protein 2 OS=Mus mus	IBP2_MOUSE	33 kDa	1	1	3	3	2.0	0.00
Fibrinogen beta chain OS=Mus musculus GN=Fgb PE=1 SV	FIBB_MOUSE	55 kDa	55	52	79	81	1.5	0.01
Ig heavy chain V regions TEPC 15/S107/HPCM1/HPCM2/I	HVM18_MOUSE (+4)	14 kDa	1	1	2	2	1.5	0.01
Ig kappa chain V-V region MOPC 173 OS=Mus musculus P	KV5AA_MOUSE	12 kDa	6	6	11	12	1.8	0.01
H-2 class I histocompatibility antigen, D-K alpha chain OS	HA13_MOUSE	41 kDa	3	3	2	2	0.7	0.01
Coagulation factor X OS=Mus musculus GN=F10 PE=1 SV=	FA10_MOUSE	54 kDa	24	26	14	13	0.6	0.01
Prolow-density lipoprotein receptor-related protein 1 OS	LRP1_MOUSE	505 kDa	7	7	5	5	0.7	0.01
Mannan-binding lectin serine protease 2 OS=Mus muscu	MASP2_MOUSE	76 kDa	14	14	9	10	0.7	0.01
Keratin, type II cytoskeletal 2 epidermal OS=Mus musculu	K22E_MOUSE	71 kDa	12	11	9	8	0.7	0.01
Ig kappa chain V-V region HP R16.7 OS=Mus musculus PE	KV5AB_MOUSE (+1)	12 kDa	9	8	17	15	1.8	0.02
Ig gamma-1 chain C region, membrane-bound form OS=N	IGH1M_MOUSE (+1)	43 kDa	54	47	20	14	0.4	0.02
Ig kappa chain C region OS=Mus musculus PE=1 SV=1	IGKC_MOUSE	12 kDa	27	31	62	54	2.0	0.02

Phosphatidylinositol-glycan-specific phospholipase D OS=	PHLD_MOUSE	93 kDa	50	53	43	42	0.8	0.02
Immunoglobulin J chain OS=Mus musculus GN=Jchain PE	IGJ_MOUSE	18 kDa	10	7	18	17	1.9	0.02
Ig heavy chain V region B1-8/186-2 OS=Mus musculus GN	HVM07_MOUSE (+1)	15 kDa	5	4	2	2	0.5	0.03
Fibulin-1 OS=Mus musculus GN=Fbln1 PE=1 SV=2	FBLN1_MOUSE	78 kDa	6	6	10	9	1.5	0.03
Apolipoprotein E OS=Mus musculus GN=Apoe PE=1 SV=2	APOE_MOUSE	36 kDa	53	60	34	38	0.6	0.03
Adiponectin OS=Mus musculus GN=Adipoq PE=1 SV=2	ADIPO_MOUSE	27 kDa	5	5	7	6	1.2	0.04
H-2 class I histocompatibility antigen, Q8 alpha chain OS=	HA18_MOUSE	37 kDa	8	7	6	5	0.7	0.04
Ig lambda-3 chain C region OS=Mus musculus GN=Iglc3 P	LAC3_MOUSE	11 kDa	4	4	5	5	1.2	0.05
Thrombospondin-4 OS=Mus musculus GN=Thbs4 PE=1 SV	TSP4_MOUSE	106 kDa	4	4	6	5	1.3	0.05
Major urinary protein 3 OS=Mus musculus GN=Mup3 PE=	MUP3_MOUSE	21 kDa	8	7	3	0	0.3	0.05
Ig mu chain C region OS=Mus musculus GN=Ighm PE=1 SV	IGHM_MOUSE	50 kDa	128	160	74	81	0.5	0.05
