# Supplementary material for: Combined Id1 and Id3 Deletion Leads to Severe Erythropoietic Disturbances
Source: PLoS One. 2016 Apr 29;11(4):e0154480. doi: 10.1371/journal.pone.0154480 (PMC4851361; doi:10.1371/journal.pone.0154480)
Supplement: S2 Table — Analysis was performed on n = 2 WT mice and n = 3 Id cDKO mice. (PDF) [file pone.0154480.s004.pdf]

S2 Table. Pathway analysis of complex 'omics data for Id cDKO bone marrow cells.

Top Diseases and Bio Functions

Diseases and Disorders

Name	p-value	# Molecules
Infectious Disease	1.08E-21 - 1.00E-03	308
Hematological Disease	9.71E-19 - 8.68E-04	214
Immunological Disease	9.71E-19 - 7.42E-04	278
Inflammatory Disease	9.71E-19 - 4.93E-04	236
Inflammatory Response	9.71E-19 - 1.28E-03	236

Molecular and Cellular Functions

Name	p-value	# Molecules
Cell Death	4.88E-29 - 1.19E-03	557
RNA Post-Transcriptional Modification	1.66E-23 - 8.67E-04	93
Protein Synthesis	1.01E-22 - 8.67E-04	252
Cellular Growth and Proliferation	1.39E-19 - 9.01E-04	510
Nucleic Acid Metabolism	7.66E-19 - 1.04E-03	163

Physiological System Development and Function

Name	p-value	# Molecules
Immune Cell Trafficking	2.24E-11 - 1.07E-03	158
Hematological System Development and Function	9.03E-11 - 1.19E-03	235
Organismal Survival	3.68E-08 - 1.02E-03	231
Embryonic Development	8.91E-07 - 1.02E-03	85
Endocrine System Development and Function	1.51E-06 - 1.51E-06	7

Top Canonical Pathways

Name	p-value	Ratio
EIF2 Signaling	3.40E-35	80/202 (0.396)
Regulation of eIF4 and p70S6K Signaling	1.11E-22	57/174 (0.328)
Protein Ubiquitination Pathway	3.37E-22	77/268 (0.287)
Mitochondrial Dysfunction	4.89E-17	47/174 (0.27)
mTOR Signaling	4.46E-16	56/209 (0.268)

Top Molecules

Fold Change up-regulated

Molecules	Exp. Value	Exp. Chart
FES	13.150	
GPR98D	13.070	
PGRMC1	8.340	
CPSF1	7.890	
LASP1	6.720	
PRMT5	6.620	
ATXN3	6.560	
IGHM	5.650	
Ugt1a7c	4.710	
Egln1D	4.630	

Fold Change down-regulated

Molecules	Exp. Value	Exp. Chart
COPE	-3.571	
ASAH1	-3.448	
FTL	-3.030	
HBB	-2.778	
MEF2D	-2.381	
CDC42	-2.273	
CTSD	-2.174	
ACP5D	-2.174	
MPP1	-2.128	
HMGCL	-2.041	

Top Upstream Regulators

Upstream Regulator	p-value of overlap	Predicted Activation State
MYC	1.15E-36	
MYCN	1.73E-23	
NFE2L2	8.07E-19	
E2F1	3.29E-08	
XBP1	4.00E-08	

Top Tox Lists

Name	p-value	Ratio
Mitochondrial Dysfunction	1.35E-16	47/136 (0.346)
Oxidative Stress	7.08E-06	17/57 (0.298)
NRF2-mediated Oxidative Stress Response	4.81E-05	40/231 (0.173)
Cardiac Necrosis/Cell Death	2.22E-04	33/191 (0.173)
Cell Cycle: G2/M DNA Damage Checkpoint Regulation	9.32E-04	12/48 (0.25)

Top Tox Functions

Assays: Clinical Chemistry and Hematology

Name	p-value	# Molecules
Increased Levels of Hematocrit	1.97E-02 - 1.97E-02	15
Increased Levels of LDH	2.47E-02 - 2.47E-02	4
Increased Levels of Albumin	9.06E-02 - 1.73E-01	2
Decreased Levels of Albumin	9.63E-02 - 6.48E-01	5
Increased Levels of Bilirubin	1.73E-01 - 1.73E-01	1

Cardiotoxicity

Name	p-value	# Molecules
Cardiac Necrosis/Cell Death	7.09E-04 - 3.16E-01	33
Cardiac Fibrosis	8.67E-04 - 5.32E-01	22
Cardiac Inflammation	2.77E-03 - 4.35E-01	10
Cardiac Damage	6.45E-03 - 5.32E-01	9
Cardiac Dilation	2.16E-02 - 5.24E-01	11

Hepatotoxicity

Name	p-value	# Molecules
Hepatocellular Carcinoma	3.85E-05 - 2.14E-01	75
Liver Hyperplasia/Hyperproliferation	3.85E-05 - 2.14E-01	76
Liver Damage	1.51E-02 - 3.78E-01	22
Liver Hepatitis	2.94E-02 - 1.00E00	11
Liver Hypoplasia	7.32E-02 - 7.32E-02	7

Nephrotoxicity

Name	p-value	# Molecules

Renal Tubule Injury	6.45E-06 - 3.32E-01	28
Renal Inflammation	8.20E-03 - 1.00E00	22
Renal Nephritis	8.20E-03 - 1.00E00	22
Kidney Failure	2.16E-02 - 5.36E-01	19
Renal Damage	5.15E-02 - 4.33E-01	13
